# Absence of a gestational diabetes phenotype in the LepRdb/+ mouse is independent of control strain, diet, misty allele, or parity

**DOI:** 10.1038/srep45130

**Published:** 2017-03-24

**Authors:** Jasmine F. Plows, XinYang Yu, Ric Broadhurst, Mark H. Vickers, Chao Tong, Hua Zhang, HongBo Qi, Joanna L. Stanley, Philip N. Baker

**Affiliations:** 1 Liggins Institute, University of Auckland, New Zealand; 2 Department of Obstetrics and Gynecology, The First Affiliated Hospital of Chongqing Medical University, Chongqing, China; 3 Canada-China-New Zealand Joint Laboratory of Maternal and Fetal Medicine, Chongqing Medical University, Chongqing, China; 4 AgResearch, Ruakura, Waikato, New Zealand; 5 College of Medicine, Biological Sciences and Psychology, University of Leicester, UK

## Abstract

Treatment options for gestational diabetes (GDM) are limited. In order to better understand mechanisms and improve treatments, appropriate animal models of GDM are crucial. Heterozygous db mice (db/+) present with glucose intolerance, insulin resistance, and increased weight gain during, but not prior to, pregnancy. This makes them an ideal model for GDM. However, several recent studies have reported an absence of GDM phenotype in their colony. We investigated several hypotheses for why the phenotype may be absent, with the aim of re-establishing it and preventing further resources being wasted on an ineffective model. Experiments were carried out across two laboratories in two countries (New Zealand and China), and were designed to assess type of control strain, diet, presence of the misty allele, and parity as potential contributors to the lost phenotype. While hyperleptinemia and pre-pregnancy weight gain were present in all db/+mice across the four studies, we found no consistent evidence of glucose intolerance or insulin resistance during pregnancy. In conclusion, we were unable to acquire the GDM phenotype in any of our experiments, and we recommend researchers do not use the db/+ mouse as a model of GDM unless they are certain the phenotype remains in their colony.

Gestational Diabetes (GDM) is defined as glucose intolerance that develops or is first recognised during pregnancy. It affects an estimated 7–18% of pregnancies in the United States, and up to 20% of pregnancies in some populations^1^,^2^. GDM is a serious concern because of the significant short-term and long-term health consequences it can have for both the mother (increased risk of surgical delivery, pre-eclampsia and type 2 diabetes in later life) and the fetus (increased risk of macrosomia, birth injury, future obesity and type 2 diabetes)^3^,^4^,^5^,^6^. The number of cases of GDM is rapidly increasing worldwide, in parallel with the obesity epidemic, making the disease a global health concern^7^. For example, in urban China, the prevalence of GDM has increased substantially from 2.3% in 1999^8^, to 17.5% in 2013, according to the IADPSG criteria^9^.

There are limited treatment options currently available to women diagnosed with GDM. These include insulin and metformin, which both have caveats attached to their use. Insulin is difficult to administer - requiring daily self-injection during pregnancy – and is associated with increased weight gain during pregnancy, which itself is known to negatively affect pregnancy outcomes^10^. Metformin has shown significant promise as a treatment for GDM and other diseases of insulin resistance, but the long-term implications of use during pregnancy remain poorly understood^11^. Because of the personal and economic impact of GDM, the fact that its prevalence is increasing, and the lack of effective treatments, much research is dedicated to understanding both the pathophysiology and treatment of the disease.

Due to ethical and logistical limitations, animal models of human disease are frequently employed in scientific research. Animal models can assist in the understanding of the pathophysiology of a disease and in the testing of potential treatments. Several animal models of GDM have been developed. These include chemical, dietary, and genetic models. However, these models have various limitations in their replication of GDM. For example, chemical methods (alloxan, streptozotocin) irreversibly destroy the β cells of the pancreas, resulting in a phenotype more reminiscent of type 1 diabetes than of GDM^12^,^13^. Dietary models, such as high fat feeding, are effective, but lack the pregnancy-specific factor of GDM^14^,^15^. Most genetic models are knockouts of genes in the pancreas, again simulating type 1 diabetes or non-pregnancy specific glucose intolerance^16^,^17^,^18^,^19^.

One genetic model of GDM - the LepRdb/+ mouse (db/+) - is considered a more representative animal model of GDM than those aforementioned. db/+ mice are heterozygous for a point mutation of the leptin receptor, which renders the receptor inactive^20^. While female mice appear normal in the virgin state, they develop glucose intolerance during pregnancy^21^,^22^,^23^,^24^,^25^,^26^,^27^. In addition, offspring of db/+ mice suffer similar consequences to children affected by GDM (obesity, diabetes in later life)^28^,^29^, and db/+ mothers that are allowed to age after giving birth also show a phenotype that is reminiscent of post-pregnant mothers afflicted by GDM^30^,^31^. As db/+ mice are the only model to spontaneously develop hyperglycemia upon the onset of pregnancy, they are a highly relevant model of GDM.

While researchers have detailed glucose intolerance, hyperglycemia, increased weight gain, increased food intake, impaired blood vessel function, and aberrant metabolic profile in db/+ mice for many years, some recent studies have reported a lack of phenotype. Harrod *et al*. (2011) published findings that indicated normal glucose tolerance in pregnant db/+ mice. The GDM phenotype could only be unmasked upon the addition of a high fat diet^32^. Similarly, Pollock *et al*. (2015) reported *improved* glucose tolerance in db/+ mice compared to WT mice, despite significant hyperleptinemia in the former^33^. Recently, we studied a potential new treatment for GDM in db/+ mice; however, we similarly did not observe glucose intolerance in the model.

The purpose of the current study was to try and determine the cause of the phenotypic discrepancy across colonies, in order to prevent further time and money wastage on a model that no longer works. We hypothesised that reasons for the loss of phenotype might include: the type of control animal used as a comparison (WT littermates of db/+ mice vs C57BL/6J), the type of diet used (standard laboratory chow or purified diet), the presence/absence of the marker misty (m) allele (a coat colour allele used in some db/+ colonies in order to allow for quick identification of genotype), and the effect of parity (i.e. is the phenotype demonstrable in multiparous animals)? These parameters were chosen as all have been shown to have an impact on the ability to observe phenotypes in genetically modified mouse models, and, in the case of maternal diet and multiparity, have also been associated with the development of GDM in human pregnancies.

These studies were carried out across two sites: the AgResearch small animal containment (SAC) facility in Waikato, New Zealand, and Chongqing Medical University in P.R. China. This allowed us to ensure that any conclusions drawn were not simply the result of an abnormality in one individual colony.

## Methods

### Study 1: db/+ mice vs WT mice born from db/+ parents vs C57BL/6J mice

#### AgResearch SAC, New Zealand

In order to determine if the type of control mouse used affects whether or not db/+ mice have comparably poor glucose tolerance, db/+ mice were compared to both WT littermates and C57BL/6J mice.

#### Animals

db/+ mice (strain B6.BKS(D)-Leprdb/J; stock number 000697) were imported from Jackson Laboratories, USA, and housed in the Small Animal Containment Unit at AgResearch, Ruakura, Waikato, New Zealand. Heterozygous (db/+) pairings were set up in order to produce both db/+ and WT female mice. Mice were housed in a light-controlled room (lights on at 0600 h, off at 1800 h) at 22 °C with 30–70% humidity, and given *ad libitum* access to AIN-93G purified diet (Research Diets, NJ, USA: 21.0% kcal from protein, 74% carbohydrate, 5% fat) and water. All animal procedures were approved by the AgResearch Animal Ethics Committee (Waikato, New Zealand) in accordance with the New Zealand Animal Welfare Act, 1999.

#### *Genotyping for the Lepr* ^*db*^ *mutation*

Genomic DNA was extracted by boiling 2 mm tail clippings in 75 μl 25 mM NaOH/0.2 mM EDTA for one hour, and adding 75 μl Tris HCl, pH 5.5^34^. PCR amplification was performed by adding 2 μl genomic DNA to 23 μl of a cocktail containing 12 μl KAPA HotStart Ready Mix (KAPA BioSystems, MA, USA), 1.2 μl forward primer (Integrated DNA Technologies, IA, USA - 5′-AGA ACG GAC ACT CTT TGA AGT CTC-3′), 1.2 μl reverse primer (Integrated DNA Technologies, IA, USA - 5′-CAT TCA AAC CAT AGT TTA GGT TTG TGT-3′), and 8.6 μl H_2_0 per reaction. Amplification was carried out in BioRad PCR machine using a PCR profile of 1 cycle at 95 °C for 3 mins; 32 cycles of 95 °C for 15 s, 60 °C for 15 s, 72 °C for 15 s, and 1 cycle at 72 °C for 1 min. The PCR product was then incubated overnight with 25 μl of a cocktail containing 1 μl RsaI restriction enzyme (New England Biolabs, MA, USA), 5 μl CutSmart buffer (New England Biolabs, MA, USA), and 19 μl H_2_0, per reaction. Finally, the product was run on 4% agarose gel and visualized with ethidium bromide. The presence of a band at 135 bp indicated a WT, two bands at 108 and 135 indicated a heterozygote (db/+), and the presence of a single band at 108 indicated a homozygote. Homozygotes were not used in the study. Genotyping was confirmed at the end of each study via an external company (Transnetyx Inc., TN, USA), in order to ensure no clerical errors had occurred during the course of the experiment.

#### Experimental protocol

WT (n = 8), db/+ (n = 8), and C57BL/6J (n = 8) female mice were weaned onto AIN-93G purified diet at three weeks of age, and remained on the diet for eight weeks. At this point, an oral glucose tolerance test (OGTT) was performed according to the method developed by Andrikopoulos *et al*.^35^. Mice were then mated with genotypically-matched males. When a copulatory plug was detected, female mice were separated and singly housed with their food consumption and weight gain monitored. This was denoted day 0.5 of gestation (GD0.5). At day 16.5 of gestation (GD16.5), another OGTT was undertaken. At day 18.5 (GD18.5), mice were fasted for 6 hours before having a tail vein blood sample taken, and were culled by cervical dislocation. Several organs including the pancreas, liver, and fat depots were removed and snap frozen, and fetuses were weighed and measured. Fetuses were also sexed, and fetal tail samples were taken for genotyping, using the same method outlined in the previous section. The blood sample was subsequently centrifuged at 10,000 rpm for 10 minutes in order to obtain plasma for insulin and leptin analysis.

#### Plasma analysis

Insulin and leptin ELISAs were carried out according to the manufacturer’s instructions (UltraSensitive Mouse Insulin ELISA [Crystal Chem #90080] and Mouse Leptin ELISA [Crystal Chem #90030]). Insulin resistance score was determined using HOMA-IR, which was calculated as follows:


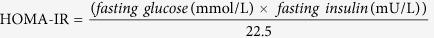


### Study 2: The effect of standard chow (rather than purified diet) on db/+ and WT mice

#### AgResearch SAC, New Zealand

In order to establish whether type of diet affects phenotype, both WT (n = 8) and db/+ (n = 8) mice were also put on a standard laboratory chow diet (autoclaved Speciality Feeds Rat and Mouse Cubes, Glen Forest, WA, AUS: 23% kcal from protein, 65% carbohydrate, 12% fat). The same procedures applied as in Study 1, except mice were weaned onto, and remained on, standard laboratory chow throughout the course of the experiment, and no pre-pregnancy OGTT was performed.

### Study 3: db/+ mice vs WT mice born from db/+ parents (with the misty allele) vs C57BL6/J mice

#### Chongqing Medical University, P.R. China

In order to establish whether the presence of the misty (*m*) coat colour allele affected whether phenotypic differences were seen across db/+ mice, similar procedures were carried out as in Study 1, except using mice with the *m* allele.

#### Animals

db/+ mice with the misty allele (Lepr^db/+^, strain B6.BKS(D)-Lepr^db^/JNju, stock number: 000697) and C57BL/6J mice (strain C57BL/6J Nju, stock number: 000664) were acquired from the Animal Research Centre of Nanjing University at 6 weeks of age to establish the db/+ and C57BL/6J colonies. Mice were housed in a light-controlled (lights on at 0800 h, off at 2000 h) at 21–25 °C with 30–70% humidity room and given *ad libitum* access to water and standard laboratory chow (^60^Co irradiated Rat & Mouse Breeder Diet 1035, Beijing HFK Bioscience, China: 23.4% kcal from protein, 71.2% carbohydrate, 5.4% fat). All animal procedures were approved by the Ethical Committee of Chongqing Medical University (Chongqing, China) in accordance with the *Chongqing Management Approach of Laboratory Animals* (Chongqing government order No. 195).

#### *Genotyping for the Lepr* ^*db*^ *mutation*

Genotyping was performed as in Study 1. In addition, the presence of the misty (m) allele in these mice confirmed the genotype. WT mice appeared dark grey in colour, while db/+ mice appeared black in colour.

#### Experimental protocol

The pups of db/+ colony mice were group housed according to genotype. At twelve-weeks of age, virgin female db/+ (n = 6), WT littermate control (n = 6) and C57BL/6J control mice (n = 6) were housed overnight with age- and genotype- matched male mice. The presence of a copulatory plug the following morning was designated as GD0.5. On GD16.5, an oral glucose tolerance test was performed as in Study 1. On GD18.5, mice were fasted for 6 hours and then culled via cervical dislocation. Blood was then collected via cardiac puncture to be used to measure fasting glucose, insulin and leptin concentrations. The uterus was removed, and fetuses and placentas dissected, weighed and measured. Fetuses were also sexed, and fetal tail samples were taken for genotyping. Maternal fat pads (gonadal, retroperitoneal, perirenal), liver and kidneys were removed and weighed.

#### Measurement of plasma insulin and leptin

Plasma concentrations of insulin and leptin were measured using mouse insulin ELISA kit (Shanghai Jianglai Biotech, Shanghai, P.R. China, #KB11459), and mouse leptin ELISA kit (Shanghai Jianglai Biotech, Shanghai, P.R. China, #KB11317), according to the manufacturer’s instructions.

### Study 4: db/+ vs WT vs C57BL6/J mice in their second pregnancy

#### Chongqing Medical University, P.R. China

In order to investigate whether a second gestation would unveil the GDM phenotype in pregnant db/+ mice, female db/+ (n = 6), WT (n = 6), and C57BL/6J control mice (n = 6) were raised and mated as in Study 3, but were allowed to litter and nurse their pups. After weaning, the dams were mated with genotypically-matched male mice again, in order to produce a second pregnancy. Body weight was measured at GD0.5 and GD18.5, and an oral glucose tolerance test was performed at GD16.5, as described in the previous studies. Maternal and pup samples were not measured/collected in this study.

### Statistical analysis

The results of all studies are presented as the mean ± standard error of the mean (SEM) for the indicated number of mice. Comparisons between groups were made using one-way ANOVA for studies 1 and 3, and Student’s unpaired t test, for studies 2 and 4. Pup growth data was analysed using three-way ANOVA, with maternal genotype, fetal genotype, and fetal sex being the three factors of interest. Statistical significance was set at *P* < 0.05. Statistical analysis and figures were generated using GraphPad Prism.

## Results

### Study 1: db/+ mice vs WT mice born from db/+ parents vs C57BL6/J mice

Study 1 was designed to establish whether the type of control animal (WT mice born of db/+ parents or C57BL6/J mice) affected whether or not a gestational diabetes phenotype was observed in db/+ mice fed purified AIN-93G diet.

#### Glucose tolerance before and during pregnancy

**Figure 1:**
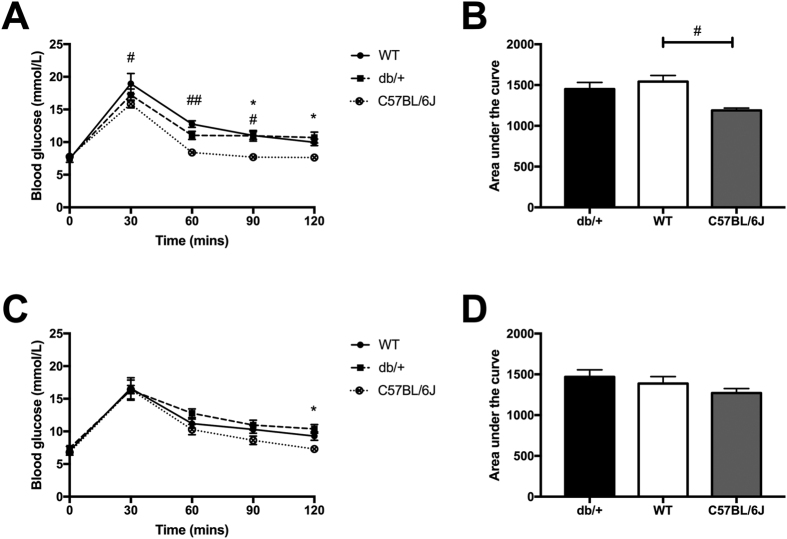
There were no consistent differences in glucose tolerance between db/+ mice and either type of control strain both prior to, or during, pregnancy. (**A**) OGTT results prior to the onset of pregnancy (12 weeks old). An asterix (*) indicates a significant difference between db/+ and C57BL/6J mice. A pound sign (^#^) indicates a significant difference between WT and C57BL/6J mice (*p < 0.05. ^##^p < 0.01). db/+ mice had significantly higher blood glucose at time = 90, and time = 120 of the glucose tolerance test, compared to C57BL/6J mice. WT mice had significantly higher blood glucose than C57BL/6J mice at every time point apart from fasting (time = 0). There were no significant differences between WT and db/+ mice. (**B**) AUC of OGTT prior to onset of pregnancy (12 weeks old). WT mice had significantly higher area under the curve than C57BL/6J mice, prior to pregnancy (^##^p < 0.01). (**C**) OGTT results at GD16.5. db/+ mice had significantly higher blood glucose at time = 120 (*p < 0.05). (**D**) AUC of OGTT at GD16.5.

There were no differences in glucose tolerance *prior to* pregnancy between WT and db/+ mice. db/+ mice had a higher blood glucose concentration than C57BL/6J mice at time = 90 (p = 0.016), and time = 120 (p = 0.026). Surprisingly, WT mice had significantly higher blood glucose than C57BL/6J mice at every time-point of the glucose tolerance test apart from at fasting (time = 0; Fig. 1A). This is reflected by a significant increase in the area under the curve (Fig. 1B; p = 0.015).

There was no difference between db/+ mice and WT mice at any time point during the oral glucose tolerance test *during* pregnancy (GD16.5) (Fig. 1C). db/+ mice had higher blood glucose compared to C57BL/6J mice at 120 minutes (p = 0.033), but not at any of the other time points. There were no differences in area under the curve (AUC) of the oral glucose tolerance test at GD16.5 across any of the groups (Fig. 1D).

#### Fasting glucose, insulin, and leptin

**Figure 2:**
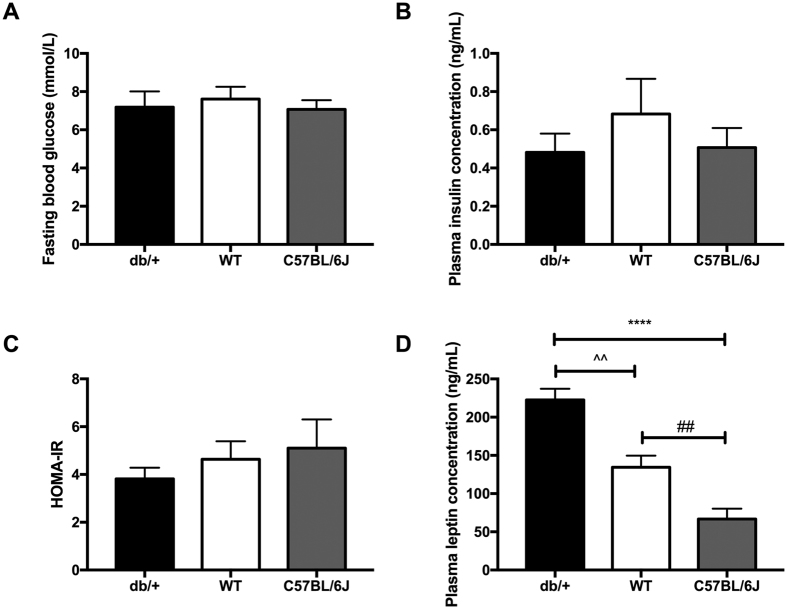
db/+ mice showed marked fasting hyperleptinemia, but no difference in glucose or insulin. (**A**) Fasting blood glucose at GD18.5. (**B**) Fasting insulin concentrations at GD18.5. (**C**) HOMA-IR score at GD18.5. (**D**) Fasting leptin concentration at GD18.5. db/+ mice had significantly higher fasting plasma leptin than both WT (^^p < 0.01) and C57BL/6J mice (****p < 0.0001). WT mice also had significantly higher plasma leptin than C57BL/6J mice (^##^p < 0.01).

There were no differences in fasting glucose (Fig. 2A) or plasma insulin (Fig. 2B) concentrations between db/+, WT, or C57BL/6J mice at GD18.5. There were also no differences in HOMA-IR across the groups (Fig. 2C). However, db/+ mice had increased fasting plasma leptin concentration compared to both WT mice (p = 0.002) and C57BL/6J mice (p < 0.0001) at GD18.5 (Fig. 2D). In addition, WT mice had increased plasma leptin compared to C57BL/6J mice (p = 0.009).

#### Maternal and fetal measurements

db/+ mice were heavier than both WT (p = 0.033) and C57BL6/J (p = 0.0005) mice at GD0.5 (Table 1). The difference between db/+ mice and C57BL/6J mice was larger than the difference between db/+ mice and WT mice. However, this weight differential did not remain at GD18.5, and there were no differences in weight gain, food intake, or energy intake over pregnancy across the groups.

db/+ mice had increased gonadal (p = 0.028) and retroperitoneal fat distribution (p = 0.001) (expressed as % body weight [BW]) compared to C57BL6/J mice, and db/+ mice also had increased retroperitoneal fat distribution compared to WT mice (p = 0.022).

Male pups were heavier than female pups in both fetal weight (p = 0.015), and placental weight (p = 0.038), but there were no other differences in fetal growth across groups (Table 2).

### Study 2: db/+ mice on standard chow vs purified diet

Study 2 was designed to establish whether feeding mice a standard laboratory chow (rather than a purified diet as in Study 1), would unmask a GDM phenotype.

#### Glucose tolerance during pregnancy

**Figure 3:**
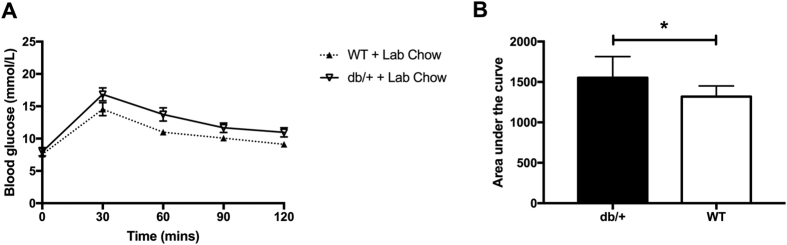
db/+ mice had a significantly larger area under the OGTT curve than WT mice in Study 2. (**A**) OGTT results at GD16.5. (**B**) AUC of OGTT at GD16.5. The asterix (*) indicates a significant difference between db/+ and WT mice in area under the curve (p < 0.05).

There was no difference between db/+ mice and WT mice on laboratory chow at any time point during the oral glucose tolerance test at GD16.5 (Fig. 3A). However, db/+ mice had a small but significantly larger area under the curve of the OGTT plot than WT mice (Fig. 3B; p = 0.049).

#### Fasting glucose, insulin, and leptin at GD18.5

**Figure 4:**
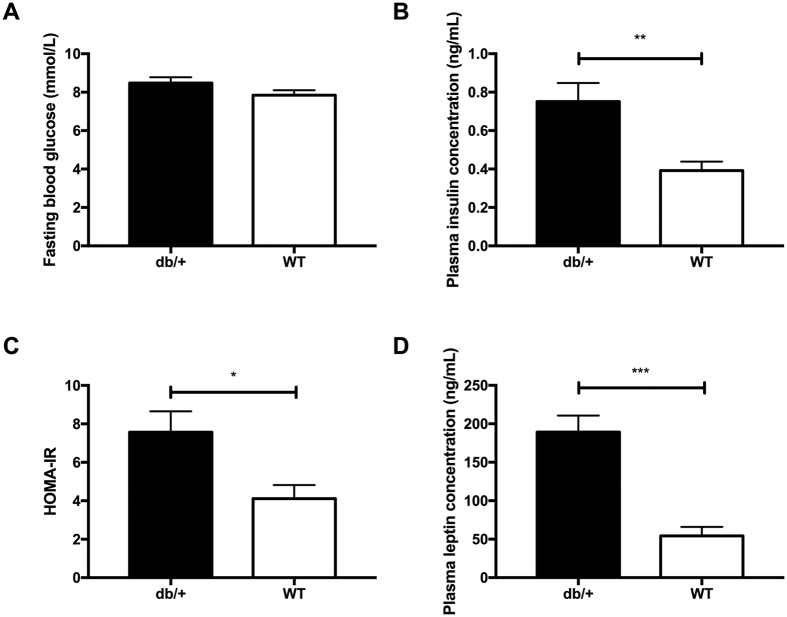
db/+ mice showed increased fasting blood glucose, insulin, HOMA-IR, and leptin compared to WT mice in Study 2. (**A**) Fasting blood glucose at GD18.5. (**B**) Fasting insulin concentrations at GD18.5. There was a significant difference between db/+ mice and WT mice in insulin concentration (**p < 0.01) (**C**) HOMA-IR score at GD18.5. There was a significant difference between db/+ and WT mice in HOMA-IR (*p < 0.05) (**D**) Fasting leptin concentration at GD18.5. There was a significant difference between db/+ and WT mice in fasting leptin concentration (***p < 0.001).

There was no difference in fasting glucose between db/+ and WT mice at GD18.5 (Fig. 4A). However, unlike the mice fed a purified diet (Study 1), there was a significant difference between db/+ and WT mice in fasting plasma insulin concentration and HOMA-IR. db/+ mice had increased fasting plasma insulin compared to WT mice (Fig. 4B; p = 0.009). They also had increased insulin resistance compared to WT mice, as measured by HOMA-IR (Fig. 4C; p = 0.039). As with the mice on purified diet (Study 1), db/+ mice were significantly hyperleptinemic compared to WT mice (Fig. 4D; p = 0.0003).

#### Maternal and fetal measurements

db/+ mice were significantly heavier than WT mice at both GD0.5 (p = 0.049) and GD18.5 (p = 0.003). However, there were no differences between the two groups in weight gain or food intake over pregnancy. As with the mice on purified diet (Study 1), db/+ mice had increased fat deposition in the gonadal (p = 0.023) and retroperitoneal (p = 0.023) depots (Table 3). Pups from WT dams were longer in crown-rump length than pups from db/+ mice (p = 0.018), but there were no other differences in fetal growth across groups (Table 4).

### Study 3: db/+ mice vs WT mice born from db/+ parents vs C57BL6/J mice (with misty allele)

Study 3 was designed to establish whether the presence of the coat colour misty (m) allele affects the development of the gestational diabetes phenotype in db/+ mice on standard chow diet.

#### Glucose tolerance during pregnancy

**Figure 5:**
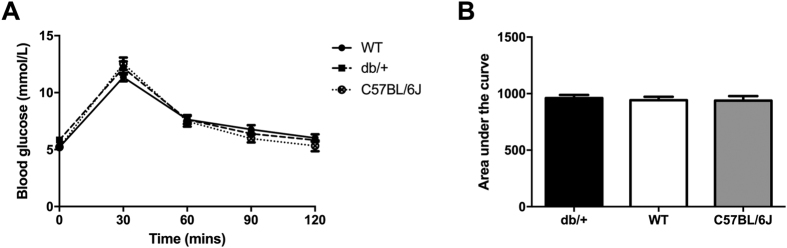
There were no differences in glucose tolerance between misty db/+ mice and either type of control in Study 3. (**A**) OGTT results at GD16.5. (**B**) AUC of OGTT at GD16.5.

At GD16.5, there was no difference in OGTT results among groups (Fig. 5A). There were also no differences in the area under the curve of the OGTT plots (Fig. 5B).

#### Fasting glucose, insulin, and leptin at GD18.5

**Figure 6:**
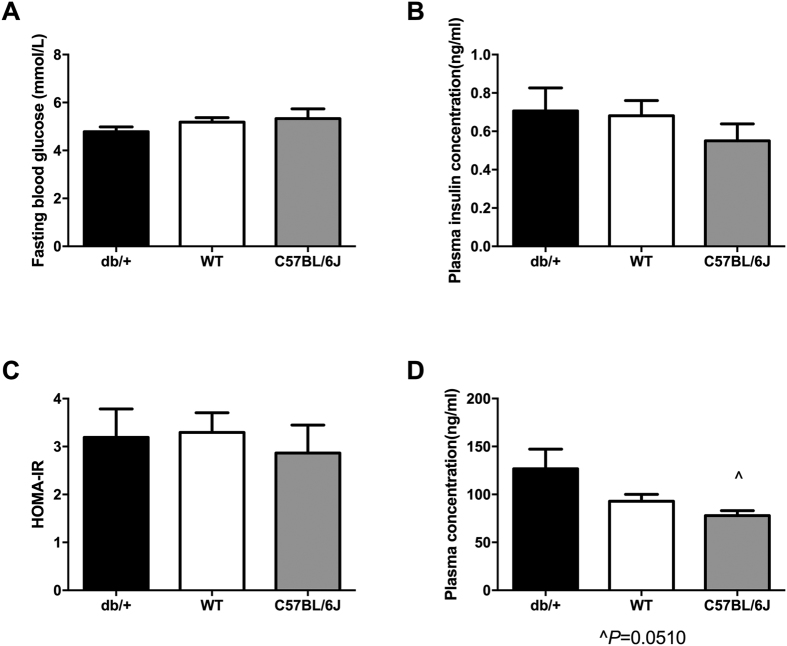
There were no differences in fasting glucose or insulin between db/+ mice and control mice in Study 3, and there was only a slight difference in leptin. (**A**) Fasting blood glucose at GD18.5. (**B**) Fasting insulin concentrations at GD18.5. (**C**) HOMA-IR score at GD18.5. (**D**) Fasting leptin concentration at GD18.5. There was almost a significant difference between db/+ and C57BL/6J mice in fasting leptin concentration (p = 0.051).

At GD18.5, fasting blood glucose and fasting plasma insulin concentrations were not significantly different across the groups. Plasma leptin levels in db/+ dams were not significantly different compared with WT dams. There was a trend towards an increase in leptin concentration when db/+ mice were compared to C57BL/6J dams (Fig. 6D; p = 0.051).

#### Maternal and fetal measurements

There were no differences in maternal weight on GD0.5, GD18.5 or weight gain during pregnancy between db/+, WT, and C57BL6/J mice. db/+ dams tended to be heavier at GD18.5 compared with WT dams, but this did not reach significance (p = 0.064).

Although there were no differences in body weight across the three groups, significant weight differences were observed in maternal fat tissue. The deposition of adipose tissue in db/+ dams was significantly increased in the gonadal (p = 0.016 vs. WT mice; p = 0.023 vs. C57BL/6J mice) and perirenal (p = 0.011 vs. WT mice; p = 0.005 vs. C57BL/6J mice) depots compared with control mice. However, retroperitoneal adipose deposition was similar across the groups. Additionally, there were no significant differences in liver or kidney weight across groups (Table 5).

Fetal growth was not significantly different across the db/+, WT, and C57BL/6J groups, even when fetuses were analysed separately according to sex and genotype (Table 6).

### Study 4: db/+ vs WT vs C57BL/6J mice in their second pregnancy

Study 4 was designed to establish whether a second pregnancy would unmask a GDM phenotype in db/+ mice fed standard chow diet.

#### Glucose tolerance during pregnancy

**Figure 7:**
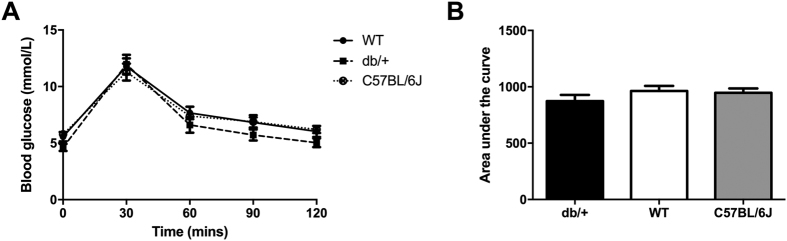
There were no differences in glucose tolerance between multiparous db/+ mice and controls in Study 4. (**A**) OGTT results at GD16.5 of a second pregnancy. (**B**) AUC of OGTT at GD16.5 of a second pregnancy.

At GD16.5, there were no differences in OGTT results across the groups (Fig. 7A). There were also no differences in the area under the curve of the OGTT plots (Fig. 7B).

#### Maternal measurements

At GD0.5, db/+ mothers were significantly heavier than WT mothers (p = 0.006) and there was a trend towards a significant increase in weight compared with C57BL/6J mothers (p = 0.051). This trend continued to GD18.5: db/+ dams was significantly heavier than WT dams (p = 0.024) but not C57BL/6J dams (p = 0.059). There were no differences in maternal weight gain over pregnancy across any of the groups (Table 7).

## Discussion

db/+ mice have been used as a model of GDM in a number of studies that span decades^21^,^36^. These animals typically present with glucose intolerance, insulin resistance, hyperglycemia, increased food intake, and increased weight gain during, but not prior to, pregnancy^22^,^23^,^24^,^25^,^26^,^27^,^30^,^31^. However, several recent studies using the model, including our own, have been unable to reproduce this phenotype^32^,^33^,^37^. The current study aimed to investigate potential reasons for the loss of a GDM phenotype in some colonies. The reason for doing so was in order to potentially salvage the model, and to avoid further valuable resources being wasted. We hypothesised that potential reasons for the lost phenotype could be the use of different control groups across studies (WT vs. C57BL6/J mice), type of diet used (AIN-93G purified diet vs. standard laboratory chow), the presence/absence of the misty (m) allele across colonies, and differences in parity.

Study 1 aimed to investigate the differences between db/+ mice and two types of controls – WT offspring of db/+ parents, and C57BL/6J mice. Most early studies in db/+ mice (in which a GDM phenotype was observed) used C57BL/6J mice as control animals^21^,^26^,^38^. However, some of the more recent db/+ studies, including our own, used WT littermates as controls^29^,^33^. This is likely due to current recommendations that knockout studies use WT offspring of the same parentage as experimental animals as controls, in order to account for epigenetic effects *in utero*^39^,^40^. We hypothesised that, while C57BL/6J mice are sufficiently different from db/+ mice to observe a difference in phenotype, WT littermates are affected by the heterozygous genotype of their parents, causing them to display a phenotype that is not significantly different from their db/+ siblings. This could create the illusion of a “missing” phenotype amongst db/+ mice, and we sought to determine if this was the case.

Study 1 found significant differences in glucose tolerance between db/+ mice and C57BL/6J mice prior to pregnancy. There were no such differences between db/+ mice and WT mice. This suggests that C57BL/6J mice have greater physiological differences compared to db/+ mice, and so are more effective control animals than WT mice. However, there were no differences in glucose tolerance across any of the three groups *during* pregnancy. Since the defining feature of GDM is glucose intolerance during pregnancy, this suggests that choice of control animal does not affect the observation of GDM in db/+ mice.

In addition to the absence of glucose intolerance during pregnancy, there were no differences between db/+, WT, and C57BL/6J mice in fasting glucose, insulin, or HOMA-IR. There was, however, a significant difference in fasting leptin between all the groups – with the largest difference observed between db/+ and C57BL/6J mice. Hyperleptinemia is to be expected due to the fact that db/+ mice have a genetic knockout of the leptin receptor. It is likely that, although the WT mice did not have the knockout themselves, epigenetic effects related to their heterozygote parentage made them more susceptible to hyperleptinemia than C57BL/6J mice^28^. What is interesting, however, is that this marked hyperleptinemia did not affect glucose tolerance or insulin resistance. It appears that an, as of yet unknown, mechanism prevented the leptin resistance from resulting in these pathologies.

The db/+ mice in Study 1 also put on more weight than both WT and C57BL/6J mice before pregnancy, but did not eat more or put on any more weight during pregnancy. This again demonstrates that the genetically-determined leptin resistance of db/+ mice was effective at causing issues *prior to* pregnancy, but for whatever reason did not produce the same effects *during* pregnancy. In addition, db/+ mice showed greater fat deposition compared to both control groups, which was likely related to the increased weight gain prior to pregnancy.

Further, no differences were observed as a result of genotype in fetal growth measurements in Study 1. Nadif *et al*. (2015) found that maternal genotype did not influence pup growth, while fetal genotype did^29^. They reported that heterozygous and homozygous db fetuses were larger than WT fetuses, as were their placentae. The fact that no such finding was observed in our study further supports a lack of phenotype in our colony.

While there were more differences between db/+ mice and C57BL/6J mice than between db/+ mice and WT mice, neither group was significantly different compared with db/+ mice. This suggests that using C57BL/6J rather than WT mice as controls does not influence whether a GDM phenotype is observed in db/+ mice. Therefore, differences in type of control strain used across studies does not appear to be the reason for discrepancies in phenotype.

Study 2 aimed to investigate whether the type of diet fed to db/+ mice – a purified or standard laboratory chow – had any effect on phenotype. Many laboratories feed their mice a standard laboratory chow, usually made from grain. This can present problems, as chow diets can be prone to batch effects, with differing levels of macro- and micronutrients across lots^41^. Purified diet, however, is kept constant, and is therefore usually preferred. There have been reports that feeding experimental animals laboratory chow versus a purified diet can yield markedly different results^42^,^43^. For this reason, we compared db/+ and WT mice fed a standard chow diet in addition to the purified diet used in Study 1, to determine if this would unmask the GDM phenotype.

There were more significant differences between WT and db/+ mice on chow diet compared to purified diet. Unlike in Study 1, there was a slight but significant difference in glucose tolerance between WT and db/+ mice during pregnancy, as assessed by the area under the OGTT curve. In addition, db/+ mice had significantly increased fasting insulin and leptin, and were insulin resistant according to HOMA-IR. As in Study 1, db/+ mice put on more weight prior to pregnancy, but not during pregnancy, and had significantly increased fat deposition. It was also found that fetuses from WT dams were significantly longer in crown-rump length than fetuses from db/+ dams, but there were no other differences in measures of fetal growth.

It is not particularly clear why the type of diet fed to db/+ mice would influence phenotype so heavily. There were no differences in food intake or calorie intake between mice on purified diet and mice on normal chow. The major differences between the purified diet and chow diet used were in fat source (saturated vs. unsaturated), fat content (15.8% vs. 12%), and fiber content (5% vs. 7.6%)^44^,^45^. However, this does not explain why a laboratory chow with lowered fat and increased fiber would result in greater glucose intolerance and insulin resistance compared with animals fed a purified diet. Indeed, increased fat and decreased fiber consumption are associated with GDM^46^, and a high fiber diet has improved the db/+ phenotype in past studies^47^, so these results are the opposite of what we would expect. Nevertheless, this finding demonstrates the importance of diet selection when testing the strength of a model.

Study 3 aimed to investigate whether the presence of the misty (m) allele within the db/+ colony affects the observation of a GDM phenotype. The misty locus is tightly linked to the LepR^db/+^ mutation on chromosome 4, and can be used as a coat colour marker that allows for quick and easy identification of genotype^36^,^48^. By about three weeks of age, WT mice appear grey in colour, while db/+ mice appear black. The misty allele was bred out of the Jackson colony in 2008, amid claims that the gene was not harmless (as previously believed) and indeed affected the db/+ phenotype^49^,^50^. For example, misty mice have been reported to exhibit stunted growth, a lack of brown fat, altered thermogenesis, impaired bone remodelling, and prolonged bleeding^49^,^50^,^51^. As these conditions affect metabolism, they are likely to influence the appearance of a GDM phenotype. We hypothesised that the misty allele is required in order to observe a GDM phenotype in db/+ mice, and that the breeding out of the allele could be responsible for the lack of GDM phenotype in recent studies.

The results of Study 3 do not support this hypothesis, as no differences in glucose tolerance were seen between db/+, WT, and C57BL/6J mice. This was the case even though the mice were fed a chow diet – the diet which most favors the development of GDM, according to Study 2. Even fasting plasma leptin concentration, which was markedly different in Studies 1 and 2, did not quite reach statistical significance in this study. However, increased GD0.5 body weight and fat deposition were again observed, further emphasising that the pregnancy-specific weight gain typical of previous db/+ models has been lost, but that hyperleptinemia still results in increased weight gain prior to pregnancy. Some studies have similarly been unable to replicate the phenotype in misty db/+ mice, including Harrod *et al*. (2011) and Bobadilla *et al*. (2010)^32^,^52^. These results suggest that the presence of the misty allele does not affect whether a GDM phenotype is observed in db/+ mice.

Finally, Study 4 aimed to test the hypothesis that multiparity would result in a GDM phenotype. Multiparity is a known risk factor for GDM^53^,^54^. Moreover, we proposed that the effects of multiparity in db/+ mice might compound the effects of aging and increased weight gain before pregnancy, and perhaps result in a more pronounced GDM phenotype^55^,^56^. However, once again we found no differences in glucose intolerance in mice in their second pregnancy. Harrod *et al*. (2011) similarly conducted glucose tolerance tests in multiparous and aged pregnant db/+ mice, and did not find differences between them^32^. Taken together, it does not appear that multiparity affects whether a GDM phenotype is seen in db/+ mice.

Of the four studies conducted, only one found glucose intolerance in db/+ mice compared to WT mice – Study 2. This difference was small and only just reached significance (p = 0.049). These mice were fed a standard laboratory chow diet rather than a purified diet. This would suggest that this diet is the best to use when using db/+ mice as a model of GDM. However, both Study 3 and Study 4 also used standard laboratory chow and saw no differences in glucose tolerance between WT and db/+ mice. This further illustrates the unpredictability of the model, and the caution that should be exercised when using it.

The combination of all four studies in this paper indicate that none of our hypotheses regarding the possible reasons underlying the loss of the db/+ phenotype were correct. Although all studies reported increased weight gain prior to pregnancy, and significant hyperleptinemia in db/+ mice, none saw the increased food intake or weight gain during pregnancy typical of previous db/+ studies. Glucose intolerance was only observed in one study, and even then it was very slight, and not replicable in our Chinese laboratory. Taken together, these results suggest that db/+ mice have become ineffective as a model of GDM.

The mice involved in these studies were acquired from either Jackson laboratories or the Nanjing University animal research center shortly before the onset of the study. At this point, it appears that the lost phenotype could be the result of adaptation within the two colonies themselves. The mice still exhibit hyperleptinemia, increased weight gain before pregnancy, and increased fat deposition, but do not show the hallmark characteristics of gestational diabetes – namely impaired glucose tolerance and increased fasting glucose during pregnancy. This suggests that the mice were able to overcome their genetically-determined leptin resistance and prevent excess weight gain and glucose intolerance during pregnancy.

There are several possible ways this adaptation could have occurred. Lack of phenotype amongst knockout mice is not uncommon, and, if genuine, is referred to as *phenotypic robustness*. There are two potential mechanisms that drive phenotypic robustness: 1) genetic buffering – in which alternate pathways for the process exist in the animal, and 2) functional complementation – in which genes can fully or partially substitute the function of another (genetic redundancy)^57^. If indeed either of these were the case in our mice, the process must have occurred independently of the development of leptin resistance, which was still present in our colonies. This process would also likely be pregnancy-specific, since pre-pregnancy weight gain and even a degree of glucose intolerance was observed in our studies prior to pregnancy, while weight gain, food intake, and glucose tolerance during pregnancy were not altered. Perhaps, in order to improve pregnancy outcomes, functional complementation and/or genetic redundancy “stepped in” to prevent overeating, excess weight gain, and glucose intolerance during pregnancy.

Another potential mechanism by which the phenotype disappeared could be environmental differences across animal facilities. For instance, there is increasing evidence to suggest that the microbiota of laboratory animals can influence phenotype^58^,^59^,^60^. While we did attempt to subvert this by conducting experiments from two different colonies in two different facilities/countries, it is possible that environmental conditions in both of our laboratories were such that we were unable to observe a GDM phenotype.

Groups that have recently reported glucose intolerance in their db/+ mice may have had an established colony that still exhibited the phenotype. These include Xing *et al*. (2015, 2016) and Nadif *et al*. (2015)^23^,^24^,^29^. These groups had previously published papers on the db/+ model, suggesting they had an established colony. A comparison of the genome/microbiome of db/+ mice from these colonies and those from the Jackson and Nanjing laboratories could reveal the source of the resistance to the phenotype.

As a final note, it is important to add that while homozygote mice were not used in these particular experiments, those that were born (approximately ¼ of the mice in the litters of heterozygote pairings) appeared to exhibit the homozygote phenotype. That is to say they were noticeably obese starting at approximately 4 weeks of age, and required more regular cage maintenance due to frequent urination. While no objective measurements were taken from these animals, it appears that there was no loss of phenotype in the homozygote db/db animals, which are commonly used as models of type 2 diabetes. Therefore, our concern about the loss of the db/+ phenotype only applies to pregnant db/+ mice, used for research into GDM.

In conclusion, it does not appear that the methodological differences explored here can explain the differing presence of the db/+ phenotype in several recent publications. We believe that the mice strains used in this study have possibly adapted to leptin resistance during pregnancy, and therefore avoid glucose intolerance. Genetic sequencing of these animals may be required in order to ascertain the source of the lost phenotype. We recommend that researchers do not use db/+ mice as a model of GDM without first establishing whether glucose intolerance is present in their particular colony.

## Additional Information

**How to cite this article**: Plows, J. F. *et al*. Absence of a gestational diabetes phenotype in the LepRdb/+ mouse is independent of control strain, diet, misty allele, or parity. *Sci. Rep.* **7**, 45130; doi: 10.1038/srep45130 (2017).

**Publisher's note:** Springer Nature remains neutral with regard to jurisdictional claims in published maps and institutional affiliations.

## Figures and Tables

**Table 1:** Maternal measurements across different control groups (Study 1).

Measurements	db/+	WT	C57BL/6J
Maternal factors			
GD0.5 body weight (g)*	24.19 ± 0.77	21.92 ± 0.55*	19.86 ± 0.28***
GD18.5 body weight (g)	38.79 ± 1.31	36.54 ± 0.80	35.77 ± 0.87
Weight gain over pregnancy (g)	13.73 ± 0.76	14.83 ± 0.73	16.00 ± 0.50
Food intake over pregnancy (g)	62.78 ± 2.02	59.86 ± 1.66	60.84 ± 1.23
Energy consumed over pregnancy (kcal)	244.84 ± 7.87	233.45 ± 6.47	237.27 ± 4.81
Gonadal fat pads weight (% BW)*	0.76 ± 0.11	0.63 ± 0.06	0.46 ± 0.03*
Perirenal fat pads weight (% BW)	0.35 ± 0.07	0.31 ± 0.05	0.21 ± 0.03
Retroperitoneal fat pads weight (% BW)**	0.28 ± 0.03	0.18 ± 0.02*	0.13 ± 0.01**
Liver weight (% BW)	3.69 ± 0.18	3.59 ± 0.20	4.10 ± 0.21
Kidney weight (% BW)	0.47 ± 0.05	0.47 ± 0.04	0.40 ± 0.02
Litter size	7.30 ± 0.67	7.67 ± 0.33	7.83 ± 0.60

An asterix (*) in the WT and/or C57BL/6J column indicates a significant difference between this group and the db/+ group. *P < *0.05*, **P < *0.01*, ***P < *0.001.*

**Table 2:** Fetal measurements across different control groups (Study 1), broken down according to maternal genotype, fetal genotype, and fetal sex.

Fetal Sex	Male Pups*					Female Pups				
Maternal genotype	WT	C57BL/6J	db/+			WT	C57BL/6J	db/+		
Fetal genotype	WT	C57BL/6J	WT	db/+	db/db	WT	C57BL/6J	WT	db/+	db/db
Weight (g)*	1.15 ± 0.05	1.16 ± 0.17	1.18 ± 0.03	1.18 ± 0.03	1.22 ± 0.01	1.03 ± 0.06	1.16 ± 0.03	1.13 ± 0.05	1.14 ± 0.04	0.96 ± 0.03
Crown-rump length (mm)	30.9 ± 0.5	30.6 ± 0.3	31.0 ± 0.4	30.9 ± 0.5	31.4 ± 0.2	30.0 ± 0.4	31.1 ± 0.3	30.5 ± 0.6	30.8 ± 0.3	29.7 ± 0.3
Abdominal circumference (mm)	25.2 ± 0.5	26.0 ± 0.3	24.9 ± 0.8	25.1 ± 0.5	25.3 ± 0.4	24.6 ± 0.6	25.7 ± 0.5	25.5 ± 0.8	25.5 ± 0.4	24.3 ± 0.3
Placental weight (g)*	0.10 ± 0.00	0.10 ± 0.00	0.10 ± 0.00	0.10 ± 0.00	0.09 ± 0.01	0.08 ± 0.00	0.09 ± 0.00	0.08 ± 0.01	0.09 ± 0.01	0.09 ± 0.01
Fetal:placental ratio	12.3 ± 0.71	11.8 ± 0.52	12.06 ± 0.64	11.72 ± 0.80	13.78 ± 1.78	13.13 ± 0.97	13.35 ± 0.34	14.05 ± 1.37	13.21 ± 1.15	10.32 ± 0.35

Male pups and placentas weighed more than female pups, indicated by the asterix *P < *0.05.*

**Table 3:** Maternal measurements across db/+ and WT females on standard chow (Study 2).

Measurements	db/+	WT
*Maternal factors*		
GD0.5 body weight (g)*	27.34 ± 1.17	23.79 ± 0.96*
GD18.5 body weight (g)**	43.06 ± 1.19	36.58 ± 1.45**
Weight gain over pregnancy (g)	15.99 ± 0.56	13.97 ± 1.49
Food intake over pregnancy (g)	76.06 ± 2.92	73.25 ± 4.66
Energy consumed over pregnancy (kcal)	254.79 ± 9.78	239.12 ± 17.50
Gonadal fat pads weight (% BW)*	1.08 ± 0.13	0.62 ± 0.09*
Perirenal fat pads weight (% BW)	0.38 ± 0.07	0.21 ± 0.07
Retroperitoneal fat pads weight (% BW)*	0.29 ± 0.04	0.16 ± 0.03*
Liver weight (% BW)	3.84 ± 0.14	3.75 ± 0.11
Kidney weight (% BW)	0.41 ± 0.01	0.40 ± 0.03
Litter size	8.5 ± 0.34	6.83 ± 0.95

An asterix (*) in the WT column indicates a significant difference between this group and the db/+ group.*P < *0.05*, **P < *0.01*.

**Table 4:** Fetal measurements across db/+ and WT mice on standard chow (Study 2), broken down according to maternal genotype, fetal genotype, and fetal sex.

Fetal Sex	Male Pups				Female Pups			
Maternal genotype	WT	db/+			WT	db/+		
Fetal genotype	WT	WT	db/+	db/db	WT	WT	db/+	db/db
Weight (g)	1.22 ± 0.08	1.17 ± 0.02	1.15 ± 0.03	1.18 ± 0.04	1.21 ± 0.03	1.06 ± 0.07	1.15 ± 0.03	1.20 ± 0.05
Crown-rump length (mm)*	31.3 ± 0.2*	29.7 ± 0.5	29.9 ± 0.4	30.5 ± 0.3	31.1 ± 0.3*	29.7 ± 0.5	29.7 ± 0.6	31.5 ± 0.8
Abdominal circumference (mm)	26.4 ± 0.8	26.0 ± 0.1	25.4 ± 0.7	25.5 ± 0.8	24.1 ± 0.7	24.7 ± 1.8	25.2 ± 1.0	26.5 ± 0.7
Placental weight (g)	0.10 ± 0.00	0.12 ± 0.01	0.12 ± 0.00	0.10 ± 0.00	0.10 ± 0.00	0.09 ± 0.01	0.11 ± 0.00	0.11 ± 0.01
Fetal:placental ratio	11.83 ± 0.97	10.08 ± 0.77	9.93 ± 0.51	11.57 ± 0.63	12.83 ± 0.63	11.2 ± 0.53	11.32 ± 0.74	11.30 ± 0.49

Pups from WT dams were longer in length than pups from db/+ dams, as indicated by the asterix. *P < *0.05.*

**Table 5:** Maternal measurements across different control groups amongst mice with the misty allele (Study 3).

Measurements	db/+	WT	C57BL/6J
*Maternal factors*			
GD0.5 body weight (g)*	20.23 ± 0.30	19.72 ± 0.35	19.80 ± 0.48
GD18.5 body weight (g)	34.13 ± 0.64	32.25 ± 0.64	32.98 ± 0.46
Weight gain over pregnancy (g)	13.93 ± 0.62	12.37 ± 0.65	13.18 ± 0.26
Gonadal fat pads weight (% BW)*	0.19 ± 0.02	0.13 ± 0.01*	0.13 ± 0.01*
Perirenal fat pads weight (% BW)**	0.14 ± 0.02	0.09 ± 0.01*	0.08 ± 0.01**
Retroperitoneal fat pads weight (% BW)	0.13 ± 0.03	0.10 ± 0.01	0.11 ± 0.01
Liver weight (% BW)	4.65 ± 0.16	4.72 ± 0.21	4.49 ± 0.15
Kidney weight (% BW)	0.78 ± 0.04	0.82 ± 0.02	0.80 ± 0.04
Litter Size	7.2 ± 0.4	6.7 ± 0.2	7.0 ± 0.4

An asterix (*) in the WT and/or C57BL/6J column indicates a significant difference between this group and the db/+ group. *P < 0.05, **P < *0.01.*

**Table 6:** Fetal measurements across different control groups amongst mice with the misty allele (Study 3), broken down according to maternal genotype, fetal genotype, and fetal sex.

Fetal Sex	Male Pups					Female Pups				
Maternal genotype	WT	C57BL/6J	db/+			WT	C57BL/6J	db/+		
Fetal genotype	WT	C57BL/6J	WT	db/+	db/db	WT	C57BL/6J	WT	db/+	db/db
Weight (g)	1.12 ± 0.01	1.13 ± 0.02	1.11 ± 0.03	1.14 ± 0.02	1.16 ± 0.04	1.12 ± 0.01	1.09 ± 0.02	1.11 ± 0.03	1.12 ± 0.02	1.15 ± 0.04
Crown-rump length (mm)	28.5 ± 0.4	28.9 ± 0.3	28.1 ± 0.9	29.4 ± 0.8	30.3 ± 0.8	29.0 ± 0.3	28.6 ± 0.3	29.4 ± 0.9	29.0 ± 0.6	28.5 ± 0.6
Abdominal circumference (mm)	25.7 ± 0.4	25.7 ± 0.4	24.2 ± 0.8	25.0 ± 0.4	26.9 ± 1.7	25.9 ± 0.3	25.7 ± 0.4	25.5 ± 1.2	24.9 ± 0.5	25.5 ± 1.2
Placental weight (g)	0.07 ± 0.00	0.08 ± 0.00	0.08 ± 0.01	0.07 ± 0.00	0.08 ± 0.00	0.07 ± 0.00	0.07 ± 0.00	0.07 ± 0.01	0.08 ± 0.00	0.08 ± 0.01
Fetal:placental ratio	15.69 ± 0.58	15.04 ± 0.32	14.77 ± 1.29	15.72 ± 0.78	15.13 ± 0.53	15.95 ± 0.42	15.10 ± 0.47	16.21 ± 1.20	14.72 ± 0.59	15.12 ± 2.34

**Table 7:** Maternal measurements after a second pregnancy.

Measurements	db/+	WT	C57BL/6J
GD0.5 body weight (g)*	24.73 ± 0.80	21.82 ± 0.25*	22.67 ± 0.48
GD18.5 body weight (g)*	37.45 ± 1.07	34.25 ± 0.56*	34.65 ± 0.73
Weight gain over pregnancy (g)	12.70 ± 0.30	12.43 ± 0.68	12.87 ± 0.52

An asterix (*) in the WT and/or C57BL/6J column indicates a significant difference between this group and the db/+ group. *P < *0.05.*
